# Epidural abscess formation after chemoradiation therapy for esophageal cancer: A case report and literature review

**DOI:** 10.1097/MD.0000000000029426

**Published:** 2022-05-27

**Authors:** Kyung Eun Shin

**Affiliations:** Department of Radiology, Soonchunhyang University Bucheon Hospital, Bucheon 14584, Republic of Korea.

**Keywords:** case report, chemoradiation therapy, complication, epidural abscess, esophageal cancer, pyogenic spondylitis

## Abstract

**Rationale:**

Esophageal cancer is one of the leading causes of death worldwide; the treatments vary according to the stage at diagnosis. Advanced esophageal cancer is usually treated by concurrent chemoradiation which is associated with complications including esophagitis, esophageal stricture or perforation, radiation pneumonitis, and/or cardiac toxicity. Herein, we describe epidural abscess, which is a very rare but severe complication that can occur after concurrent chemoradiation therapy for advanced esophageal cancer.

**Patient concerns:**

A 75-year-old man developed a fever during concurrent chemoradiation therapy for advanced esophageal cancer, which progressed to neurological deficit and paraplegia. Enhanced chest computed tomography and C-spine magnetic resonance imaging were performed.

**Diagnosis:**

Chest computed tomography revealed a poorly enhanced necrotic change in the cervical esophageal cancer, with mottled dirty material and fluid collection. C-spine magnetic resonance imaging revealed a prevertebral abscess with pyogenic spondylitis at the C6–T2 level. In addition, an anterior epidural abscess at the C6–7 level compressed the spinal cord.

**Interventions:**

The patient underwent emergency anterior cervical discectomy and decompression corpectomy.

**Outcomes:**

: After surgery, the neurological symptoms gradually improved.

**Lessons:**

Pyogenic spondylitis with an epidural abscess is a rare but life-threatening complication that can develop after concurrent chemoradiation therapy for advanced esophageal cancer. Rapid, accurate diagnosis and prompt surgical treatment are important to ensure a favorable long-term prognosis and a good quality of life.

## 1 Introduction

Esophageal cancer is common, and is usually treated with concurrent chemoradiation therapy in advanced stages. However, this therapy is associated with several complications including esophagitis, esophageal stricture or perforation, and radiation pneumonitis,^[1–3]^ all of which are readily diagnosed radiologically or based on the clinical symptoms. However, one rare but life-threatening complication can easily be missed, namely epidural abscess with pyogenic spondylitis. These abscesses are not readily apparent; nonspecific symptoms gradually worsen, resulting in severe complications such as paraplegia. It is important that clinicians are aware of the possibility of an epidural abscess; familiarity with chest computed tomography (CT) and C-spine magnetic resonance imaging (MRI) findings is essential. We present a case of epidural abscess with pyogenic spondylitis in a 75-year-old male receiving concurrent chemoradiation therapy for advanced esophageal cancer.

## 2 Case presentation

**Figure 1:**
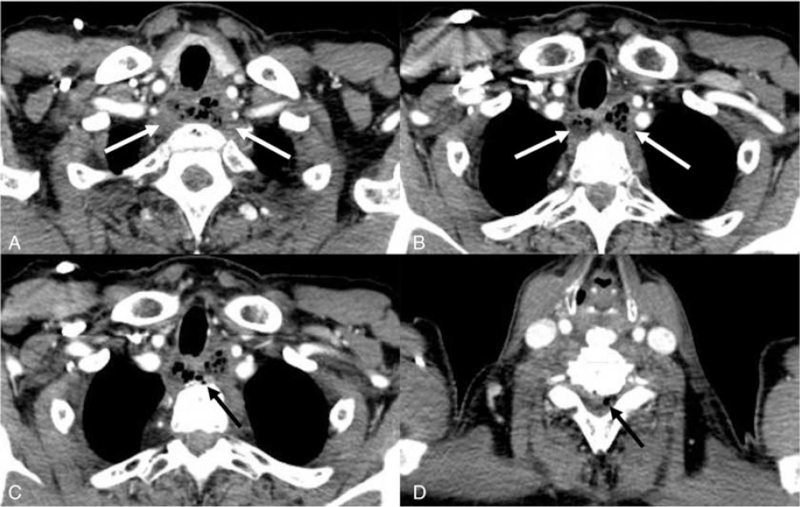
(A and B) Contrast-enhanced chest computed tomography (CT) revealed a diffuse, poorly enhanced esophageal lesion with necrosis and a mottled appearance (arrow), although there was no obvious esophageal defect or perforation. (C) On reviewing the chest CT scans after neurological symptoms appeared, small amount of free air (arrow) was apparent in the prevertebral space (D); a small amount of free air (arrow) was noted in the spinal canal.

This retrospective case report conformed to the ethical guidelines of the Declaration of Helsinki and was approved by the Institutional Review Board of the Soonchunhyang University Bucheon Hospital, and the patient provided consent for publication of this case report. He was endoscopically diagnosed with invasive cervical esophageal squamous cell carcinoma (clinical stage = T4N1M0). He had no history of hypertension, diabetes mellitus, or coronary vascular disease. He had a 52 pack-year smoking history and experienced difficulty in swallowing. He received concurrent chemoradiation therapy (5-fluorouracil, cisplatin; and 6900 cGy of radiation). Two weeks after his seventh round of chemotherapy, he was admitted with a high fever (37.8°C) and chills. On admission, his blood pressure was 110/70 mm Hg, the pulse was 79/min, and the respiratory rate was 20/min. Laboratory tests revealed neutrophilia (white blood cells, 15,570/μL; neutrophils, 13,240/μL), anemia (hemoglobin, 9.2 g/dL), mild thrombocytosis (platelet count, 422,000/μL), a high erythrocyte sedimentation rate (120 mm/h), and an elevated C-reactive protein level (13.02 mg/dL). Gram-positive cocci (*Staphylococcus constellatus*) were detected in blood culture. Contrast-enhanced chest CT revealed a poorly enhanced necrotic change of the cervical esophageal cancer, with mottled dirty material and fluid collection. There was no obvious esophageal perforation or fistula (Fig. 1A and B). Intravenous antibiotic cefepime therapy (2 g/dose every 12 hours) was started to counter the gram-positive bacteremia.

**Figure 2:**
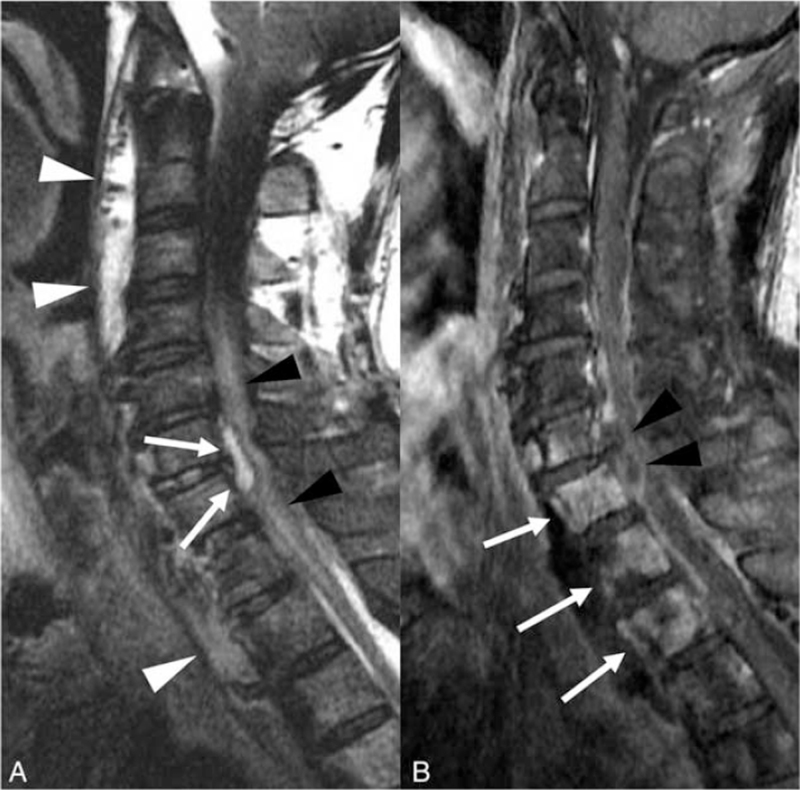
(A) The sagittal fat-saturated T2-weighted C-spine magnetic resonance imaging (MRI) shows anterior epidural fluid (arrow) in C6–7 displaces the thecal sac posteriorly. The spinal cord exhibits a diffuse, high signal intensity (black arrows heads), attributable to compression by the abscess. Also, a diffuse prevertebral abscess from C2 to T3 is apparent on the T2-weighted image (white arrowheads). (B) The sagittal T1-weighted enhanced C-spine MRI shows rim enhancement on epidural fluid collection (black arrowheads), suggesting an epidural abscess. The vertebral bodies (C6–T2) exhibit high signal intensity on the T2-weighted image and enhancement (arrows) on the T1 weighted-image, compatible with pyogenic spondylitis.

Seven days after admission, he developed motor weakness of the upper extremities, especially of the left index finger, along with yellowish sputum and an elevated body temperature (39.2°C). Two days later, paraplegia and neck stiffness were apparent. The prior chest CT images were reviewed to better understand these symptoms. Free air was identified in the prevertebral space, and focally in the spinal canal (Fig. 1C and D), but no esophageal perforation or wall defect was apparent. Under the suspicion of an epidural abscess or neurological involvement in esophageal cancer, we scheduled a contrast-enhanced C-spine MRI. Fat-saturated T2-weighted imaging (WI) revealed an anterior epidural abscess at the C6–7 level, which triggered spinal cord displacement and compression, and a cord signal change (Fig. 2A). Mildly diffuse, heterogeneous high-intensity signals arose from the vertebral bodies and disc spaces from C6 to T2. Contrast-enhanced T1-WI revealed enhancement of the vertebral bodies at levels C6–T2, compatible with pyogenic spondylitis (Fig. 2B). Fat-saturated T2-WI revealed fluid in the prevertebral space; this was somewhat enhanced on T1-WI, suggesting a prevertebral abscess that had formed in the cancer bed and then spread to the intervertebral disc and spinal canal.

We performed emergency incision and drainage for esophageal lesion and C6–7 anterior cervical discectomy and decompression corpectomy for epidural abscess. Pus-like fluid from esophageal abscess into the epidural space was found. After surgery, the patient confirmed that neurological symptoms have gradually improved. We also inserted a percutaneous endoscopy gastrostomy catheter into the stomach for nutrition. The abscess gradually decreased on follow-up chest CT. However, the patient died 1 year later from esophageal cancer with metastases to the liver and lungs.

## 3 Discussion

Esophageal cancer is the eighth most common cancer worldwide.^[4]^ The prognosis is relatively poor because most cases are locally advanced and/or distantly spread at diagnosis.^[1,4]^ Advanced esophageal cancers, which are unresectable, are usually treated by concurrent chemoradiation to improve quality of life and life expectancy.^[5–7]^ However, esophageal irradiation can cause esophagitis (16%–63% of cases), infections (2%–30%), pneumonitis (2%–18%), cardiac toxicity (3%–19%), and esophageal fistulas and strictures (8%)^[1–3]^ depending on the radiation dose, dose fractionation, treatment volume, and technique. Chemotherapeutic agents such as 5-fluorouracil can exacerbate radiation side-effects.^[3]^ Most radiation-induced complications are relatively predictable at diagnosis and thus can be controlled. However, some complications are life-threatening. One of the most severe side-effects is an epidural abscess with pyogenic spondylitis. Only a few cases have been reported on epidural abscess complications in esophageal cancer patients who underwent chemoradiation therapy followed by esophageal stent insertion or balloon dilatation for stricture (Table 1) and they have shown to be fatal if not managed properly at an early stage.^[8–10]^ To the best of our knowledge, our case is the first report on epidural abscess formation after chemoradiation therapy only without any procedures for esophageal cancer in English literature.

**Table 1:** Summary of published cases of epidural abscess complication on esophageal cancer patients.

Author	Age (y)	Sex	Tx Hx for esophageal cancer	Symptoms	Complication	Treatment
Li et al^[8]^	53	M	Chemoradiation therapyEsophageal stent insertion for the stricture	Fever, upper back painParaplegia in lower extremity	C7 to T4 pyogenic spondylitisT1 to T6 epidural abscess	T1 to T6 laminectomyAbscess drainage and decompression
Herrmann et al^[10]^	71	M	Chemoradiation therapyEsophageal dilation and stent insertion for the stricture	Back pain, feverUpper and lower extremity weaknessDifficulty in urination	Pyogenic spondylitisPosterior epidural abscess from C7 to lumbar spine	C7 corpectomyC6-T1 anterior fusionEpidural abscess drainage at C7-T1
Janssen et al^[9]^	64	F	SurgeryChemoradiotherapy, esophageal dilation for the stricture	Tetraparesis	C4 to C7 epidural abscessPrevertebral abscessDiscitis	C4 to C6 spondylodesis laminectomyEpidural abscess drainage

Hx = history, Tx = treatment.

The pathogenesis of epidural abscess in our case was first caused by necrosis and infection of the esophageal mucosa, then the necrosis and infection of esophagus spreads into the disc, vertebral body, and finally epidural space. This spread is accelerated by radiation therapy which triggers spinal osteonecrosis and mucosal deficits, resulting in reduced resistance to infection.^[11,12]^

The three primary symptoms of epidural abscesses are fever, back pain, and neurological deficit. These symptoms do not always manifest together (only 37% of cases exhibited all three).^[13]^ Their presentation is usually sequential; in terms of the neurological symptoms, back pain, radiating nerve-root pain, and motor weakness are followed by paraplegia (usually in the later stages). The speed of progression is very variable; appropriate and prompt diagnosis of an epidural abscess is challenging.^[14]^

Our patient initially complained of a high fever, which was managed with antibiotics under the suspicion of an abscess at the site of esophageal cancer. A few days later, a neurological deficit developed in the upper arm. On reviewing the chest CT scans, focal free air was noted around the prevertebral space and the spinal canal, although there was no obvious esophageal perforation. Thus, it was assumed that bacteria had spread into the disc space, spine, and epidural space. However, the treatment decision was delayed because C-spine MRI was performed only when paraplegia developed; small air bubbles in the prevertebral space and spinal canal evident on the chest CT scans were not initially detected.

Enhanced spinal MRI is the gold standard for diagnosing epidural abscess. On MRI, such an abscess presents as a bulging contoured lesion under the dura mater of the spinal canal, which is iso- or hypointense on T1-WIs and hyperintense on T2-WIs. After gadolinium enhancement, peripheral enhancement (inflammatory tissue) is seen around a non-enhancing focus (necrotic tissue).^[13]^ MRI localizes the abscess and can reveal the involvement of adjacent organs. This greatly aids surgical planning and postoperative evaluation.^[13]^

However, if a clinician is unaware that an epidural abscess can form in esophageal cancer patients on chemoradiation therapy, spinal MRI may be delayed. It is important to check for epidural abscess if the symptoms worsen, especially after treatment with intravenous antibiotics. Delayed epidural abscess treatment can result in irreversible spinal cord injury (because of cord compression). It is important to thoroughly examine chest CT scans before scheduling spinal MRI. The goal is to identify small free-air pockets in the prevertebral space and spinal canal, even if no esophageal perforation or fistula is apparent.

An epidural infection can cause spinal cord injury directly (via mechanical compression) or indirectly (via vascular occlusion of septic thrombophlebitis).^[14]^ Early and accurate diagnosis, and appropriate treatment, are essential to avoid life-threatening morbidity. Delayed surgery (by >3 days) after neurological symptoms first appear may lead to irreversible spinal cord infarction.^[15]^ Most retrospective studies recommend surgical drainage, decompressive laminectomy, and debridement of infected tissues, combined with systemic antibiotics.^[14]^ Such antibiotics should be selected based on culture susceptibility tests and prescribed for at least 6 weeks. Neurological signs and symptoms, and any sign of sepsis, should be closely followed-up, and regular imaging should be scheduled after treatment initiation.

In conclusion, an epidural abscess and pyogenic spondylitis can develop in patients with advanced esophageal cancer on chemoradiation therapy. Although the condition is very rare, it is potentially life-threatening and can cause irreversible neurological sequelae. Therefore, checking for epidural abscess is important during early diagnosis, especially in patients with focal free air around the prevertebral space and spinal canal on chest CT, or a progressive neurological deficit. Enhanced C-spine MRI is the gold standard for diagnosing epidural abscess. Early diagnosis, appropriate decompressive surgery, and abscess drainage promote a good prognosis.

## Author contributions

**Conceptualization:** Kyung Eun Shin.

**Data curation:** Kyung Eun Shin.

**Investigation:** Kyung Eun Shin.

**Writing – original draft:** Kyung Eun Shin.

**Writing – review & editing:** Kyung Eun Shin.
